# Cardiopulmonary and Immune Alterations in the Ts65Dn Mouse Model of Down Syndrome and Modulation by Epigallocatechin-3-Gallate-Enriched Green Tea Extract

**DOI:** 10.3390/pharmaceutics17111366

**Published:** 2025-10-22

**Authors:** Birger Tielemans, Sergi Llambrich, Laura Seldeslachts, Jonathan Cremer, Hung Chang Tsui, Anne-Charlotte Jonckheere, Nora Fopke Marain, Mirko Riedel, Jens Wouters, Julia Herzen, Bartosz Leszczyński, Erik Verbeken, Jeroen Vanoirbeek, Greetje Vande Velde

**Affiliations:** 1 Biomedical MRI Unit, Department of Imaging and Pathology, KU Leuven, 3000 Leuven, Belgium; sergi.llambrich@kuleuven.be (S.L.); laura.seldeslachts@kuleuven.be (L.S.); jens.wouters@gmail.com (J.W.); greetje.vandevelde@kuleuven.be (G.V.V.); 2 Translational Research Center in Gastrointestinal Disorders, Department of Chronic Diseases, Metabolism and Ageing, KU Leuven, 3000 Leuven, Belgium; 3 Laboratory of Respiratory Diseases and Thoracic Surgery (BREATHE), Department of Chronic Diseases, Metabolism and Ageing, KU Leuven, 3000 Leuven, Belgiumjeroen.vanoirbeek@kuleuven.be (J.V.); 4 Allergy and Clinical Immunology Research Group, Department of Microbiology, Immunology and Transplantation, KU Leuven, 3000 Leuven, Belgium; annecharlotte.jonckheere@kuleuven.be; 5 Research Group Biomedical Imaging Physics, Department of Physics, Technical University of Munich, 85748 Garching, Germany; mirko.riedel@tum.de (M.R.); julia.herzen@tum.de (J.H.); 6 Institute of Materials Physics, Helmholtz-Zentrum Hereon, 21502 Geesthacht, Germany; 7 Department of Medical Physics, Faculty of Physics, Astronomy and Applied Computer Science, M. Smoluchowski Institute of Physics, Jagiellonian University, 30-034 Kraków, Poland; bartosz.leszczynski@uj.edu.pl; 8 Morphology and Molecular Pathology Section, University Hospital Leuven, 3000 Leuven, Belgium; erik.verbeken@kuleuven.be

**Keywords:** Down syndrome, Ts65Dn mouse model, heart and lung development, modulation by green tea extract enriched in epigallocatechin-3-gallate, cardiovascular and immune alterations

## Abstract

**Background/Objectives**: Cardiovascular and pulmonary diseases are leading comorbidities n individuals with Down syndrome (DS). Although clinically well described, preclinical models fully characterizing these cardiopulmonary alterations are lacking. Our objective is to characterize the cardiopulmonary and immunological phenotype in a commonly used DS mouse model, the Ts65Dn mice, and investigate the modulatory effects of green tea extract enriched in epigallocatechin-3-gallate (GTE-EGCG); **Methods**: Treatment started at embryonic day 9 and continued until postnatal day (PD) 180. Mice were longitudinally monitored using micro-computed tomography, and structural, functional, and immunological alterations were evaluated at PD210 to determine the persistent effects of GTE-EGCG administration; **Results**: Ts65Dn mice displayed normal structural lung development and presented with right ventricular hypertrophy and reduced B-cell lymphocytes, indicating that this model may find applications in immunological respiratory research specific to the context of DS. GTE-EGCG administration induced transient lung immaturity, persistent decreases in lung function, and airway hyperreactivity, while normalizing arterial and right ventricular morphology and partially restoring B-cell lymphocyte numbers; **Conclusions**: These findings underscore the dual nature of EGCG modulation, both beneficial and adverse, and highlight the importance of a multiorgan, holistic approach when evaluating therapeutic interventions in DS models.

## 1. Introduction

Down syndrome (DS) is the most common genetic cause of intellectual disability [1]. In addition to neurological and cognitive deficits, individuals with DS present with a spectrum of abnormalities affecting most organs and organ systems [2]. People with DS are prone to cardiopulmonary disorders, including impaired pulmonary vascular growth, pulmonary hypertension (PH) [3,4], congenital heart diseases (CHD), chronic airway obstruction [5,6], and a compromised immune system [7,8,9]. These structural, functional, and immunological alterations result in high mortality and morbidity rates for DS individuals [10]. Although interest in DS-associated immune dysfunction has increased in recent years [11,12,13], the underlying mechanisms linking immune, pulmonary, and cardiac development and function remain incompletely understood [14]. Understanding the development and modulation of these deficits through animal models may help identify new prevention strategies.

Animal models are essential in such research as they offer a valuable tool to investigate the development of respiratory, cardiopulmonary, and immunological disorders in a controlled experiment. Because orthologs of human chromosome 21 (Hsa21) genes are distributed across mouse chromosomes 10, 16, and 17, fully replicating the complete genotype of DS in mice is challenging. The Ts65Dn model is one of the first viable trisomic mouse models for DS and has been used in preclinical studies for nearly 30 years. It remains the most commonly used due to its long-standing availability and extensive multisystemic characterization. The Ts65Dn model is trisomic for about two-thirds of the Hsa21 orthologs [15,16,17] and manifests some DS features, including craniofacial malformations, brain dysmorphology, altered bone micro-architecture, cardiac anomalies, and cognitive impairment [18,19,20]. However, Ts65Dn mice also carry 46 non-Hsa21 orthologous genes from Mmu17 that are unrelated to DS [21,22]. More recent models, such as Ts66Yah or TcMAC21, achieve a more complete trisomy of Hsa21 orthologs and may better recapitulate specific aspects of DS [23,24,25]. Nevertheless, a comprehensive and holistic understanding of DS-reminiscent pulmonary, cardiac, and immunological development in the Ts65Dn or any other mouse model of DS is currently lacking.

Currently, pharmacological treatment using green tea extract (GTE) has been tested in preclinical research as well as in the clinical situation [26,27,28,29]. GTE is rich in catechins as epigallocatechin-3-gallate (EGCG), epigallocatechin (EGC), epicatechin-3-gallate (ECG), and epicatechin (EC) [30]. Publication of a rescuing effect of GTE-EGCG on cognition, recognition, and working memory in Ts65Dn mice, as well as in children and young adults with DS [29,31,32], fueled the interest in GTE-EGCG in the DS community and beyond. Ever since, a diversity of preclinical studies has been published with a variety of experimental designs, including differences in concentration, duration, or developmental stage at which GTE-EGCG was administered, resulting in contradicting results, reporting both positive and negative treatment effects [33,34,35,36]. Since GTE enriched with EGCG is a non-regulated freely available dietary supplement, variations in GTE and EGCG composition make it difficult to compare among studies [30,37]. Therefore, further research to assess the safety, optimal dosage, and timing, as well as potential harmful effects on all body organs preclinically, is warranted when GTE-EGCG is administered for cognitive enhancements.

Additionally, EGCG-rich catechins and green tea polyphenol extracts containing EGCG exhibit antiatherogenic and antioxidative properties, potentially benefiting the cardiovascular system [38,39,40,41]. Through its known interaction with vascular endothelial growth factor (VEGF) and repressing activity of nuclear factor of activated T-cells (NFATc) signaling pathways, it is possible that EGCG had a modulatory effect on the cardiac system [42,43,44]. While numerous studies have explored the cardioprotective effects of EGCG in various models, these investigations have not been conducted in the context of DS-like trisomy [45,46]. As such, the effects of EGCG modulation on the lung, heart, and immune system in DS remain unexplored.

In this study, we aimed to (1) comprehensively characterize cardiopulmonary and immunological alterations in the Ts65Dn mouse model of DS using a holistic multiorgan approach that integrates longitudinal micro-computed tomography (µCT), echocardiography, lung function testing, histopathology, and immune profiling, and (2) assess the impact and safety of prenatal chronic GTE-EGCG administration, including the assessment of persistent effects after treatment discontinuation.

## 2. Materials and Methods

### 2.1. Ethics

This study was approved by the institutional animal ethics committee of KU Leuven (P120/2019; date of approval: 27 August 2019). All animal experiments were carried out in compliance with national and European regulations. Mice were kept in a conventional animal facility with controlled pathogen-free environmental conditions in individually ventilated cages in a biosafety level 2 facility, with free access to food and water.

### 2.2. Animal Model

Ts65Dn (B6EiC3Sn-a/A-Ts (1716)65Dn) females and B6EiC3Sn.BLiAF1/J males (refs. 005252 and 003647), were obtained from the Jackson Laboratory (Bar Harbor, ME, USA) and crossed within the next six months to obtain F1 trisomic Ts65Dn (TS) mice and euploid wild-type littermates (WT) that were used throughout the experiment. The date of conception (E0) was determined when a vaginal plug was present. Mice were genotyped at postnatal day 1 (PD1) by PCR from tail snips according to the protocol in [47] and allocated in groups according to their genotype, sex, and treatment after weaning at PD21 (see Supplemental Table S1) [47]. Mice were housed in individually ventilated cages (IVC cages, 40 cm long × 25 cm wide × 20 cm high) and separated by sex and litter, with a maximum of 5 mice per cage, and maintained under a strict 12 h light/dark schedule in controlled environmental conditions of humidity (50–70%) and temperature (22 ± 2 °C) with food and water supplied ad libitum. The bedding was refreshed every other day and sufficient nesting material was provided.

### 2.3. GTE-EGCG Treatment

**Figure 1:**
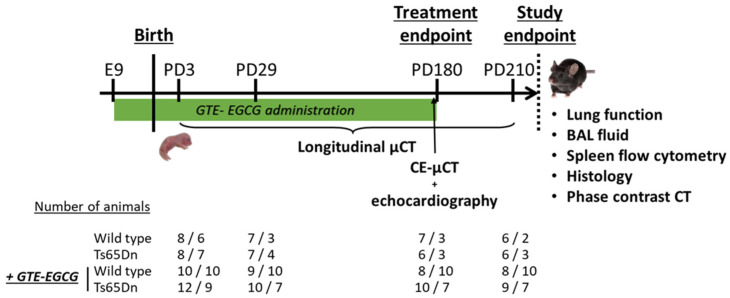
Schematic overview of experimental design. GTE-EGCG was administered via drinking water starting at embryonic day 9 (E9) and until PD180. After birth, mice were scanned using µCT at postnatal day (PD) 3, PD29, PD180, and PD210. Body weight was measured at each timepoint. At treatment endpoint, PD180, a contrast-enhanced µCT (CE-µCT) and echocardiographic analysis were performed, after which GTE-EGCG administration was discontinued. Lung function measurements, immune profile, histological samples, and phase-contrast CT were performed at study endpoint (PD210). Four groups were considered, which resulted in a number of animals after birth indicated as number of females/males at each timepoint. GTE-EGCG = green tea extract–epigallocatechin-3-gallate.

Green tea extract enriched in epigallocatechin-3-gallate (GTE-EGCG) was administered to half of the pregnant dams via the drinking water starting on embryonic day 9 (E9) [26,48]. The solution was prepared daily to ensure stability and efficacy, with GTE-EGCG powder (Mega Green Tea Extract, #00954, Life Extension, Fort Lauderdale, FL, USA) dissolved in drinking water at a final concentration of 0.09 mg EGCG/mL. This concentration corresponds to an estimated intake of approximately 30 mg EGCG/kg/day, based on an average early adult mouse body weight of 20 g and a daily water intake of ~6 mL, estimated by our own measurements. Water intake was monitored in each cage throughout the study to ensure constant exposure. The EGCG content in the capsules was calculated using previous compositional analysis (53.6% EGCG, 12.5% epigallocatechin [EGC], 9% epicatechin gallate [ECG], and 4.5% epicatechin [EC]) [48]. After weaning at PD21, the GTE-EGCG solution with the same concentration of EGCG was provided ad libitum to the mice via the drinking water up to PD180, when the treatment was discontinued (Figure 1).

### 2.4. Experimental Design

Thirteen pregnant dams were randomly divided in a non-treatment (six litters) and treatment-regimen (seven litters) group. After giving birth, the newborn pups were scanned with µCT at postnatal day (PD) 3, PD29, and at 6 months (PD180), when a contrast-enhanced µCT (CE-µCT) was performed to study vasculature and heart. This timepoint also marked the treatment endpoint, after which the administration of GTE-EGCG was discontinued. At study endpoint (PD210), the mice were scanned using µCT and sacrificed with an intraperitoneal injection of sodium pentobarbital (70 mg/kg body weight, Doléthal, Vetoquinol n.v., Niel, Belgium) for study endpoint measurements (Figure 1). The selected timepoints—PD3, PD29, and PD180—were chosen to enable a longitudinal assessment of developmental progression, from early postnatal stages (PD3), through early adulthood (PD29), to full maturity (PD180). The treatment endpoint at PD210 was designed to assess potential long-lasting effects after a 30-day post-treatment washout. Mouse body weight was recorded at each of these timepoints. Variations in mouse numbers between the experiments are either due to exclusion of data due to insufficient scan quality e.g., due to movement artifacts, or occasional mouse death upon technical issues during scanning leading to hypothermia and death.

### 2.5. Longitudinal Lung Imaging Using µCT

Mice at PD3, PD29, PD180, and PD210 were anesthetized by inhalation of 1.5–2% isoflurane in 100% oxygen and scanned using a dedicated in vivo µCT scanner (Skyscan 1278, Bruker µCT, Kontich, Belgium). At PD3; scans were acquired using 35 kVp X-ray source voltage, 500 μA current, a 0.5 mm aluminum filter and an exposure time of 180 ms per projection. At PD29, PD180, and PD210, scans were acquired using a: 55 kV X-ray source voltage, 700 μA current, a 1 mm aluminum filter, 80 ms exposure time per projection, and projections at 1° increments over a total angle of 360°, producing reconstructed 3D datasets with 50 μm isotropic voxel size. Software provided by the manufacturer (NRecon (v1.7.3.1), DataViewer (v1.5.6.2), and CTan (v1.18.8.0), Bruker, Kontich, Belgium) was used to reconstruct, visualize, and process μCT data as described previously [49,50,51]. Quantification of mean lung density, non-aerated lung volume, aerated lung volume, and total lung volume was carried out for a volume of interest (VOI) covering the lung, manually delineated on the coronal μCT images, avoiding the heart and main blood vessels. The threshold used to distinguish aerated from non-aerated lung tissue volume was manually set at −493 Hounsfield units (HU) and kept constant over all datasets.

### 2.6. Contrast-Enhanced Cardiac µCT

At PD180, a pre- and post-contrast-enhanced µCT (CE-µCT) scan was acquired to visualize heart and blood vessels. First, a pre-CE-µCT scan was acquired with similar parameters as the µCT scan, except that 20 projections per view were acquired, retrospectively time-based sorted, resulting in a reconstructed 3D dataset corresponding to the different phases of the breathing and cardiac cycle (4D) with a scanning time of 12 min. After tail vein injection with a blood pool contrast agent (Exitron nano 12,000, Milteny Biotec, Bergisch Gladbach, Germany), a second scan was acquired which allowed comparison of density changes between pre- and post-contrast scans to extract a biomarker for pulmonary vascular development in the range between fixed thresholds of −345 HU to 385 HU. The data we report here represents the end of the expiratory phase. Reconstruction, analysis, and rendering of 3D images was performed using NRecon, CTan, DataViewer, and CTvox (v3.3.0 r1403) software (Bruker, Belgium). Adobe Photoshop 2022 was used to enhance image contrast and overall sharpness.

### 2.7. Echocardiographic Analysis

For functional cardiac analysis, echocardiography was performed in parallel with CE-µCT at PD180 (treatment endpoint) in mice anesthetized by inhalation of 2% isoflurane. Animals were placed in a supine position on a heating pad to maintain the core body temperature between 37.5–37.7 °C, measured using a rectal probe, to assess the anesthesia depth and to prevent anesthesia-induced hypothermia. Electrocardiogram (ECG) recordings were performed to monitor the heart and breathing rate. 2D M-mode echocardiography and tissue and pulsed wave Doppler imaging were performed using a MS400 (18–38 MHz) transducer connected to a Vevo 2100 echocardiograph (Visualsonics, Toronto, ON, Canada). Left ventricle (LV) internal diameters were measured at end-diastole and end-systole (d and s adjuncts, respectively). LV ejection fraction (EF) was calculated as described [52]. In combination with registered heart rate and stroke volume (SV), the cardiac output (CO) was calculated. Left ventricular filling was assessed by pulsed wave Doppler trans-mitral flow tracings (gate size 0.29 mm and Doppler angle −25°), including E, A, isovolumic contraction time, aortic ejection time, isovolumic relaxation time, and mitral valve deceleration time, just above the tip of the mitral valve leaflets using an apical view. Systolic peak wave, E′, and A′ were measured with tissue Doppler imaging (gate size 0.29 mm and Doppler angle 0°) at the lateral mitral annulus using an apical view. To assess diastolic function, E/A, E/E′, E′/A′, and E/E′/SV ratios were calculated. Using the Pulse Wave Doppler mode, the right ventricle (RV) and pulmonary valve (PV) function were assessed, including the pulmonary arterial (PA) acceleration time (PAT), the PA ejection time (PET), and PV velocity–time integral (PV VTI). At least three stable cardiac cycles were averaged for all measurements.

### 2.8. Pulmonary Lung Function Measurements

Lung function measurements were performed at study endpoint (PD210) using the flexiVent FX system (SCIREQ, EMKA Technologies, Montreal, QC, Canada) equipped with flexiWare 7.6 software and a negative pressure forced expiration (NPFE) FX1 module as previously described [53]. Briefly, mice were terminally anesthetized with an intraperitoneal injection of sodium pentobarbital (70 mg/kg Doléthal, Vetoquinol n.v., Niel, Belgium). Via a tracheotomy, mice were quasi-sinusoidally ventilated with a tidal volume of 10 mL/kg and a frequency of 150 breaths/min to mimic spontaneous breathing. At the start of each measurement, two successive deep inflations were applied to maximally inflate the lungs to a pressure of 30 cm H_2_O. Next, a deep inflation was performed to determine the inspiratory capacity (IC). Forced oscillation perturbation (FOT) was initiated using the 3 s long, broadband FOT ‘Quick Prime-3′ (QP3) with a frequency between 1 and 20.5 Hz, yielding data on the central airway resistance (Rn), tissue damping (G), tissue elasticity (H), and tissue hysteresivity (eta = G/H). Next, a NPFE (negative pressure-driven forced expiration) perturbation was performed to measure the peak expiratory flow (PEF), forced vital capacity (FVC), and forced expiratory volume in 0.1 s (FEV_0.1_). From these extracted parameters, the Tiffeneau index (FEV_0.1_/FVC) was calculated to have an estimate of the obstruction in the smaller airways. Reported values are the average of three accepted measurements for each individual data point. All values were corrected for body weight and presented as such.

Airway hyperreactivity was measured by extracting the airway resistance (Rn) after inhalation of increasing concentrations of methacholine (0, 1.25, 2.5, 5, 10, 20, and 40 mg/mL). After each concentration, the QP3 perturbation was performed 5 times, spread over 2 min. If the coefficient of determination of a QP3 perturbation was lower than 0.90, the measurement was excluded and not used to calculate the average. Rn measures were plotted against the methacholine concentration, and the area under the curve was calculated to perform statistical analysis.

### 2.9. Immunological Analysis

For quantification of systemic immune cells, blood was collected after lung function measures retro-orbitally. A total of 50 µL blood was used for determining whole blood cell counts using the Advia Hematocounter (Siemens Healthineers, Forchheim, Germany).

For collection of the pulmonary immune cells, the lungs were lavaged in situ, 3 times, with 0.7 mL of sterile saline (0.9% NaCl), and the recovered bronchoalveolar lavage (BAL) fluid was pooled. Cells were counted using a Bürker hemocytometer (Brand GmbH, Wertheim, Germany) (total cell count) and the BAL fluid was centrifuged (1000× *g*, 10 min). To obtain the differential cell count, 250 μL of the resuspended cells (100,000 cells/mL) were spun (300× *g*, 6 min) (Cytospin 3; Shandon, TechGen, Zellik, Belgium) onto microscope slides, air-dried, and stained (Diff-Quik^®^ method; Medical Diagnostics, Düdingen, Germany). For each sample, 250 cells were counted for the number of macrophages, eosinophils, neutrophils, and lymphocytes.

The spleen of each mouse was collected for dedicated systemic immune cell analysis and kept in cold RPMI-1640 medium with Glutamax (Cat: 61870–010, Invitrogen, Waltham, MA, USA). Cell suspensions were obtained by pressing the spleen through a cell strainer (100 µm) (BD Bioscience, Franklin lakes, NJ, USA) and rinsing with 10 mL of tissue culture medium. After centrifugation (1000× *g*, 10 min), cells were counted and resuspended as 10^7^ cells/mL in complete tissue culture RPMI-1640 medium containing 10% heat-inactivated fetal bovine serum (#15290018, Life Technologies, Carlsbad, CA, USA) and 10 mg/mL streptomycin/penicillin (#15140122, Life Technologies Carlsbad, CA, USA). If the cell numbers were lower than 10^6^, 100 μL of complete tissue culture medium was added for suspension. After the suspension, 500,000 cells were immunostained with anti-CD3+ (APC), anti-CD4+ (APC-Cy7), anti-CD8+ (PerCP-Cy5.5), and anti-CD25+ (PE), or received a single staining with anti-CD19+ (PE)-labeled antibodies (BD Biosciences, Franklin lakes, NJ, USA). Percentages of labeled cells were determined by an LSR Fortessa flow cytometer (BD Biosciences, Belgium). Data was analyzed using FlowJo software (v10.6.2, TreeStar, Inc., Ashland, OR, USA). Lymphocytes were gated into B cell (CD19+), T cell (CD3+), T helper cell (CD3+, CD4+), regulatory T cell (CD3+, CD4+, CD25+), and cytotoxic T cell (CD3+, CD8+) populations.

### 2.10. Histopathological and Histochemical Analysis

During lung harvest for microscopic histological analysis, the left lungs were instilled with 4% formaldehyde, inflated at a pressure of 25 cm fluid column and used for histopathological examination. Sagittal sections of 5 µm were cut and stained with hematoxylin–eosin (H&E) and Sirius red and evaluated by an experienced pathologist in a blinded manner. Airspace enlargement was quantified in 10–15 fields per lung, taken structurally randomized, by measuring the mean linear intercept (Lm). The pulmonary vascular wall thickness was analyzed on section stained with an Elastica von Gieson staining, by measuring the inner and outer diameter of 12 random arteries in each mouse [54,55].

Each heart was removed, immersion-fixed in paraformaldehyde 4% for 24 h and embedded in paraffin en bloc. Sections of 5 µm thick transmural tissue were taken from the septal and lateral wall at a mid-ventricular level. Microscopic analysis was performed with a Zeiss Axio Imager ML light microscope (Zeiss, Oberkochen, Germany) measuring septal thickness, LV lumen, maximal diameter of the RV (Dmax), and the lateral wall thickness of the RV and LV at mid-ventricular level. Cardiomyocyte hypertrophy was measured on hematoxylin–eosin (H&E)-stained sections, by measuring the Lm value [56,57]. The number of cardiomyocytes transected by a reference line of 500 µm at ×200 magnification was counted on 10 fields per sample and then averaged.

### 2.11. Phase-Contrast µCT

Quantitative phase-contrast µCT scans (PC-µCT) were performed at PETRA III, DESY, Hamburg, Germany at the imaging beamline P05 operated by the Helmholtz-Zentrum Hereon, Geesthacht, Germany. The samples were embedded in paraffin wax for scanning, where a beam energy of 20 keV and an exposure time of 65 ms was used with an effective pixel size of 1.2 µm.

Further details about the setup and scan protocol can be found in [58]. Phase retrieval was computed using unified modulated pattern analysis [59]. Before tomographic reconstruction using filtered back-projection, a twofold binning was applied.

### 2.12. Statistical Analyses

The data were analyzed using GraphPad Prism (version 9.01. GraphPad Software Inc., La Jolla, CA, USA). The data are presented as individual values with median or as mean with standard deviations (SD). For analysis we were interested in the differences between genotype and the effect of treatment. Therefore, we performed a statistical test to answer each of the five research questions. We compared WT and Ts65Dn mice to evaluate if there was a genotype effect; WT and WT treated mice to evaluate if there was a treatment effect in the WT background; Ts65Dn and Ts65Dn treated mice to evaluate if there was a treatment effect in the trisomic background; WT and Ts65Dn treated mice to evaluate if the treatment was rescuing the DS phenotype; and WT treated and Ts65Dn treated mice to evaluate genotype-specific treatment effects. These pairwise comparisons were defined a priori based on our specific hypotheses.

Data on longitudinal µCT-derived biomarkers (mean lung density, total lung volume, non-aerated lung volume, and aerated lung volume) were analyzed using a mixed-effects model with Geisser–Greenhouse correction. Since time always resulted in a significant difference, as mice grew and their lung volume increased accordingly over time, we focused on differences in the growth patterns as reflected by the interaction effect, which is indicated by ‘+’ when significant.

Pairwise comparison between groups at single timepoints was performed as described in [48,60]. To account for variability within and among groups, normality and homoscedasticity tests were performed before the corresponding statistical test was used. Normality was assessed for each comparison using the Shapiro–Wilk test and homoscedasticity was assessed using an F-test. Mice identified as outliers by the ROUT test [48] with a Q (maximum desired False Discovery Rate) of 1% in combination with a technical or biological reason were excluded from the analysis. When data were normally distributed and variances were equal, we performed a standard (Student’s) *t*-test. When data were normally distributed but variances were unequal, we performed a Welch’s *t*-test and presented the data as mean and standard deviation. In case data were not normally distributed but standard deviations were similar, we performed a Mann–Whitney test and expressed data as median with interquartile range. In case data were not normally distributed and the standard deviations were not similar, we performed a two-sample Kolmogorov–Smirnov test. For all tests, statistical significance was assumed when the *p*-value < 0.05. A summary of normality, homoscedasticity, and statistical tests for pairwise comparison is provided in Supplemental Table S2.

To investigate a potential interaction between genotype and treatment effect, we performed a two-way ANOVA. If an interaction between genotype and treatment was present, this is indicated in the graphs with # depending on the level of significance.

## 3. Results

### 3.1. Tracing Body Weight Development Showed a Genotype Effect upon GTE-EGCG Administration

**Figure 2:**
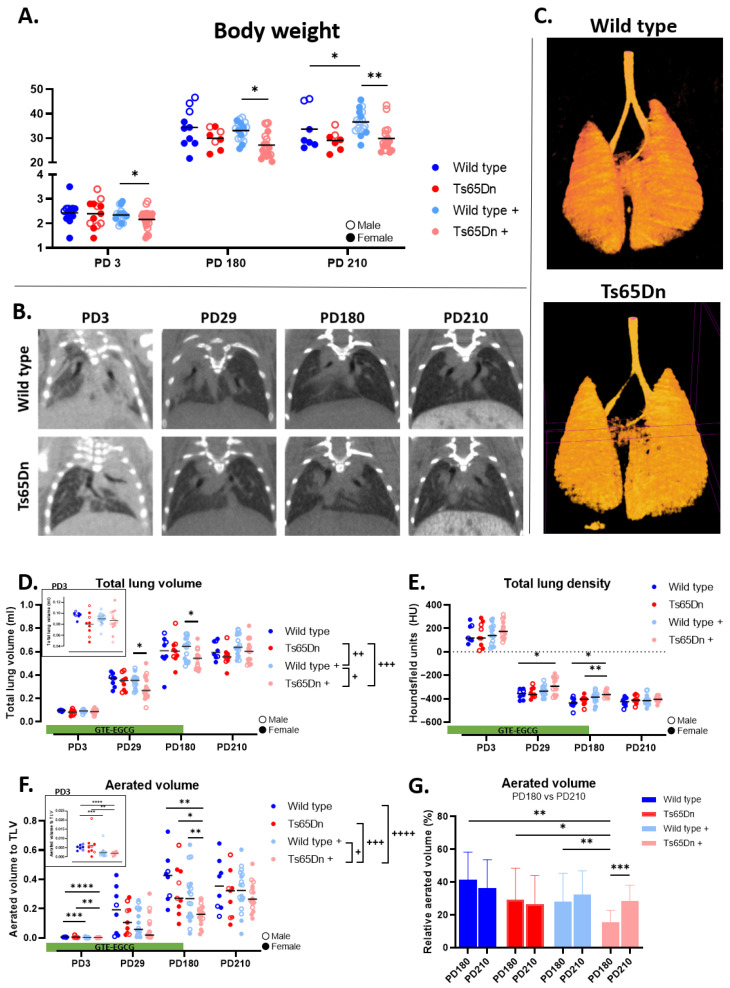
Analysis of body weight and structural pulmonary phenotype throughout development. (**A**) Body weight measurements over postnatal development in untreated and treated WT and Ts65Dn mice. (**B**) Representative tomographic lung images of WT and Ts65Dn mice at PD3, PD29, PD180, and PD210. (**C**) Three-dimensional (3D) visualization of aerated lung volumes acquired with µCT. µCT-derived biomarkers including: (**D**) total lung volume in mL, (**E**) total mean lung density in Hounsfield units (HU), and (**F**) aerated lung volume corrected according to total lung volume and expressed as percentage (%) were plotted for WT (blue line), Ts65Dn (red line), GTE-EGCG-treated WT (light blue line), and Ts65Dn (light red line) mice. (**G**) Detailed analysis of aerated volumes at treatment endpoint (PD180) and study endpoint (PD210). Data are presented as (**D**–**F**) individual values along with group median and (**G**) mean with SD. Distributions are each compared by pairwise comparison including unpaired *t*-test, Welch’s *t*-test, Mann–Whitney test, or Kolmogorov–Smirnov test (* *p* < 0.05, ** *p* < 0.01, *** *p* < 0.001, **** *p* < 0.0001) at each specific timepoint and by a mixed-effects analysis across timepoints (**D**–**F**); + *p* < 0.05; ++ *p* < 0.01; +++ *p* < 0.001, ++++ *p* < 0.0001. Males (open dot) and females (full dot) were presented accordingly. WT untreated n = 8 (2 males), Ts65Dn untreated n = 9 (3 males), WT treated n = 18 (10 males), and Ts65Dn treated n = 16 (7 males). No outliers were identified.

First, we investigated the body growth of mice by assessing the body weight of both WT and Ts65Dn mice throughout their development with and without GTE-EGCG treatment. At any developmental stage, no genotype differences were observed between WT untreated and Ts65Dn untreated mice (Figure 2A).

Prenatal administration of GTE-EGCG resulted in a lower body weight at PD3 in Ts65Dn treated mice compared to WT treated mice. At treatment endpoint, at PD180, the Ts65Dn mice still presented with a lower body weight compared to WT treated mice (Figure 2A). At this adult stage, Ts65Dn treated mice also showed a reduced body weight compared to WT untreated mice (Figure 2A). After treatment discontinuation, both WT and Ts65Dn treated mice showed a slight increase in body weight resulting in a higher body weight of WT treated mice compared to WT untreated mice (Figure 2A). The mixed-effects analysis to report differences in growth pattern demonstrated an equal development in body weight between genotypes and treatment groups.

### 3.2. Characterization of the Structural and Functional Pulmonary Phenotype of Ts65Dn Mice Throughout Development and Modulatory Effects of GTE-EGCG

#### 3.2.1. Structural Lung Development Is Similar in WT and Ts65Dn Mice

We tracked lung development of euploid and trisomic mice longitudinally using high-resolution in vivo µCT from postnatal day 3 (PD3) to PD210. Qualitative analysis revealed no obvious differences in lung structure between WT and Ts65Dn mice (Figure 2B). 3D reconstruction of the aerated lung volume further comfirmed comparable lung structure between WT and Ts65Dn mice at study endpoint (Figure 2C). Quantitative analysis of macrostructural lung biomarkers, including total lung volume, showed normal lung growth in both genotypes (Figure 2D). Total lung densities decreased over time in both WT and Ts65Dn mice indicating a similar lung maturation (Figure 2E). Alveolar growth and airway maturation showed similarly increased aerated lung volumes in both genotypes, supporting equal lung maturation (Figure 2F). Comparison of µCT biomarkers demonstrated similar lung growth and maturation in WT and Ts65Dn mice. (Figure 2D,E).

**Figure 3:**
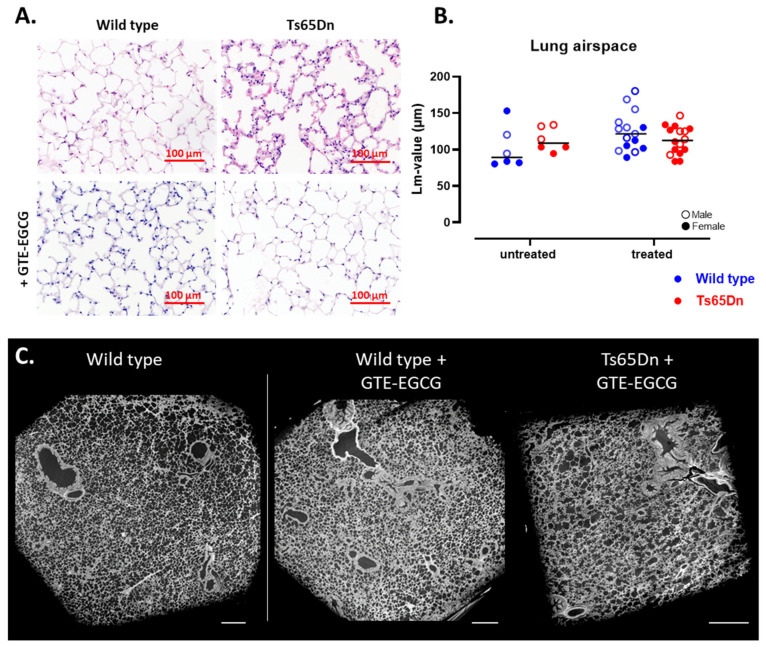
Visualization and microstructural analysis of lung parenchyma. (**A**) Histological hematoxylin and eosin (H&E) staining (magnification ×20) of WT and Ts65Dn mice with and without GTE-EGCG administration, scale bar = 100 µm. (**B**) Quantification of alveolar airspace (Lm-value) from lung parenchyma sections shown in (**A**) Data are presented for each individual mouse along with group medians. Distributions were each pairwise compared by unpaired *t*-test. WT (blue) and Ts65Dn (red) with and without treatment of GTE-EGCG, males (open dot) and females (full dot) were presented accordingly. WT untreated n = 6 (2 males), Ts65Dn untreated n = 6 (3 males), WT treated n = 15 (9 males), and Ts65Dn treated n = 16 (7 males). No outliers were identified. (**C**) Representative high-resolution phase-contrast µCT images of 3D morphology of lung parenchyma at alveolar level (scale bar = 250 µm; voxel size 2.5 µm). Full reconstructions are included in Supplemental Videos.

Microstructural measurements were conducted to assess alveolar complexity and growth by analysis of airspace enlargement using the mean linear intercepts (Lm) of lung parenchyma on histological sections (Figure 3A). Quantification of airspace enlargement demonstrated comparable Lm value’s between WT and Ts65Dn mice, thereby indicating comparable alveolar complexity and suggesting similar lung maturation and lung development in both genotypes (Figure 3B). In addition, representative qualitative analysis of 3D high resolution PC-µCT images supported similar findings (Figure 3C). These images are intended as qualitative investigations of the lung microstructure down to alveolar level, full 3D datasets are provided (see Supplemental Videos S1–S3). Taken together, the lung macro- and microstructural phenotype development is similar in both genotypes.

#### 3.2.2. GTE-EGCG Administration Alters the Lung Maturation Trajectory

Next to extracting baseline lung development, we investigated the modulatory effect of prenatal administration of GTE-EGCG on lung development in euploid and trisomic mice. Longitudinal µCT scans revealed the macrostructural lung development up to six months of age. The mixed-effects analysis across timepoints demonstrated a decrease in lung growth indicated by a significant reduction in total lung volume in the GTE-EGCG treated groups compared to untreated Ts65Dn mice (Figure 2D). Longitudinal analysis showed that Ts65Dn mice had a larger response to GTE-EGCG as the decrease in total lung volume was more pronounced in trisomic mice (Figure 2D). When investigating single timepoints, we observed that the total lung density of Ts65Dn mice administered with GTE-EGCG was larger at PD29, indicating lower lung capacities (Figure 2E). This difference in lung density increased throughout development until treatment endpoint at PD180. Similarly, at treatment endpoint, lung maturation, indicated by the aerated lung volume, was significantly lower in Ts65Dn mice compared to both WT groups (Figure 2F). These results highlight a larger response of trisomic mice to GTE-EGCG administration. Interestingly, after GTE-EGCG discontinuation, no variations in mean lung densities and aerated lung volume were observed at the study endpoint (Figure 2E,F). Comparing the aerated lung volumes at the treatment endpoint and study endpoint showed a significant increase in Ts65Dn mice (*p* = 0.0009; 0.1559 ± 0.07 vs. 0.2838 ± 0.097) 30 days after GTE-EGCG discontinuation (Figure 2G).

Histological analysis of lung parenchyma at the study endpoint revealed comparable alveolar development and lung airspace between WT and Ts65Dn treated mice (Figure 3A,B). This observation, supported by µCT-extracted aerated lung volumes, confirms that GTE-EGCG administration did not induce persistent microstructural changes in alveolar complexity and lung parenchyma. Furthermore, high-resolution PC-µCT demonstrated similar lung maturation (Figure 3C, Supplementary Videos S1–S3). Overall, these findings suggest that while the trajectories of pulmonary maturation are delayed in GTE-EGCG-treated trisomic mice, at the study endpoint, after discontinuation of GTE-EGCG administration, lung airway and parenchymal maturation was similar between treated and untreated adult Ts65Dn and WT mice.

#### 3.2.3. WT and Ts65Dn Littermates Present with Equal Pulmonary Lung Function and Airway Reactivity

**Figure 4:**
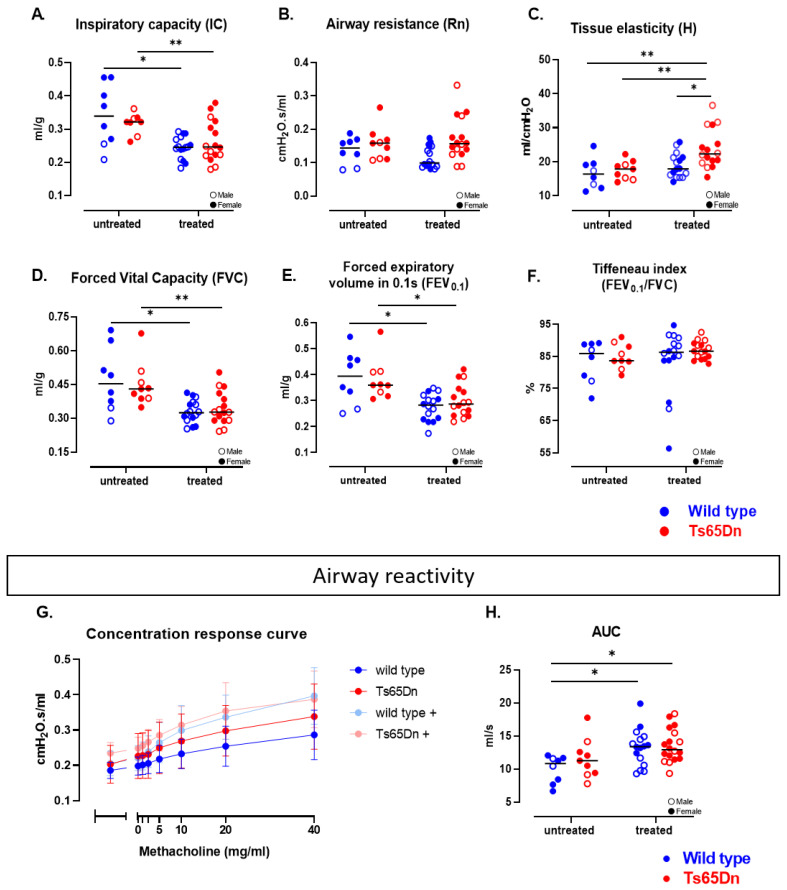
Baseline lung function analysis and airway responsiveness at study endpoint in WT and Ts65Dn mice with and without GTE-EGCG modulation. (**A**) Inspiratory capacity (IC), (**B**) central airway resistance (R), (**C**) tissue elasticity (H), (**D**) FVC, (**E**) FEV_0.1_, and (**F**) Tiffeneau index (FEV_0.1_/FVC). (**G**) Airway resistance was measured in response to increasing methacholine aerosol challenges (0–40 mg/mL) in all four groups and presented as group mean ± SD and a mixed-effects analysis was performed across timepoints. (**H**) Calculations of AUC of (**G**) were presented for all groups. All data (except **G**) are presented as individual values along with group means and distributions each pairwise compared by unpaired *t*-test (**H**), Welch’s *t*-test (**C**), and Kolmogorov–Smirnov test (**A**,**B**,**D**–**F**) (* *p* < 0.05, ** *p* < 0.01). Interactions between genotype and treatment were analyzed using a two-way ANOVA and males (open dot) and females (full dot) were presented accordingly. WT untreated n = 8 (2 males), Ts65Dn untreated n = 9 (3 males), WT treated n = 16 (9 males), and Ts65Dn treated n = 16 (7 males). Two outliers (WT treated) were identified and excluded due to technical issues and mortality of the mice during measurements.

Lung function measurements were conducted at study endpoint to investigate the genotype effect on pulmonary function and airway reactivity. Trisomic and WT mice exhibited comparable inspiratory capacity (Figure 4A), consistent with the µCT structural biomarkers. WT and Ts65Dn mice had similar airway resistance, tissue elasticity, forced vital capacity (FVC), forced expiratory volume in the first 0.1 sec (FEV_0.1_), and FEV_0.1_/FVC ratio (Figure 4B–F), indicating the absence of restrictive or obstructive lung disease. Subsequently, WT and Ts65Dn mice also showed comparable airway reactivity to increasing methacholine concentrations (Figure 4G–H), concluding that euploid and trisomic mice have a similar functional lung phenotype.

#### 3.2.4. GTE-EGCG Reduces Inspiratory Capacity and Sensitizes for Airway Hyperreactivity

At study endpoint, lung function analysis showed that GTE-EGCG administration resulted in lower inspiratory capacity in both WT and Ts65Dn mice (Figure 4A) while airway resistance was unaffected (Figure 4B). Treated Ts65Dn mice, but not WT mice, exhibited higher tissue elasticity (Figure 4C) In both treated groups, GTE-EGCG reduced the FVC and FEV_0.1_ (Figure 4D,E). A similar Tiffeneau index suggests no persistent obstructive phenotype in the smaller airways (Figure 4F).

Furthermore, we examined the impact of GTE-EGCG on non-specific airway reactivity using methacholine. GTE-EGCG administration induced a persistent and enhanced hyperreactive response in both euploid and trisomic mice compared to the untreated group (Figure 4G,H).

Taken together, although there were no genotype differences in lung function, GTE-EGCG administration reduced inspiratory capacity, FVC and FEV_0.1_ and increased tissue elasticity in Ts65Dn mice. Treatment with GTE-EGCG increased the airway reactivity persistently in both treated groups even after a 30-day treatment discontinuation.

In summary, the overall characterization of Ts65Dn mice regarding structural and functional lung development using our longitudinal µCT analysis revealed no genotype-dependent differences in lung maturation during a seven-month follow-up. Lung function measures at the study endpoint confirmed similar lung function in Ts65Dn and WT mice. 2D lung histology and 3D PC-µCT supported these findings at the microstructural level. Remarkably, GTE-EGCG administration during the treatment period reduced lung maturation, particularly in trisomic mice. Despite the normalization of lung maturation after treatment discontinuation, persistent decreases in lung function were observed in both genotypes. Additionally, GTE-EGCG administration induced a hyperreactive phenotype in the airways, which persisted even after a 30-day treatment discontinuation period.

### 3.3. Characterization of the Structural and Functional Cardiovascular Phenotype of Ts65Dn Mice and Impact of GTE-EGCG Modulation

We next assessed the pulmonary vasculature and cardiac structure and function using contrast-enhanced µCT (CE-µCT), echocardiography, and histological examination of the pulmonary vasculature and heart.

#### 3.3.1. Ts65Dn Mice Present with a High Variation in Arterial Vessel Thickness

**Figure 5:**
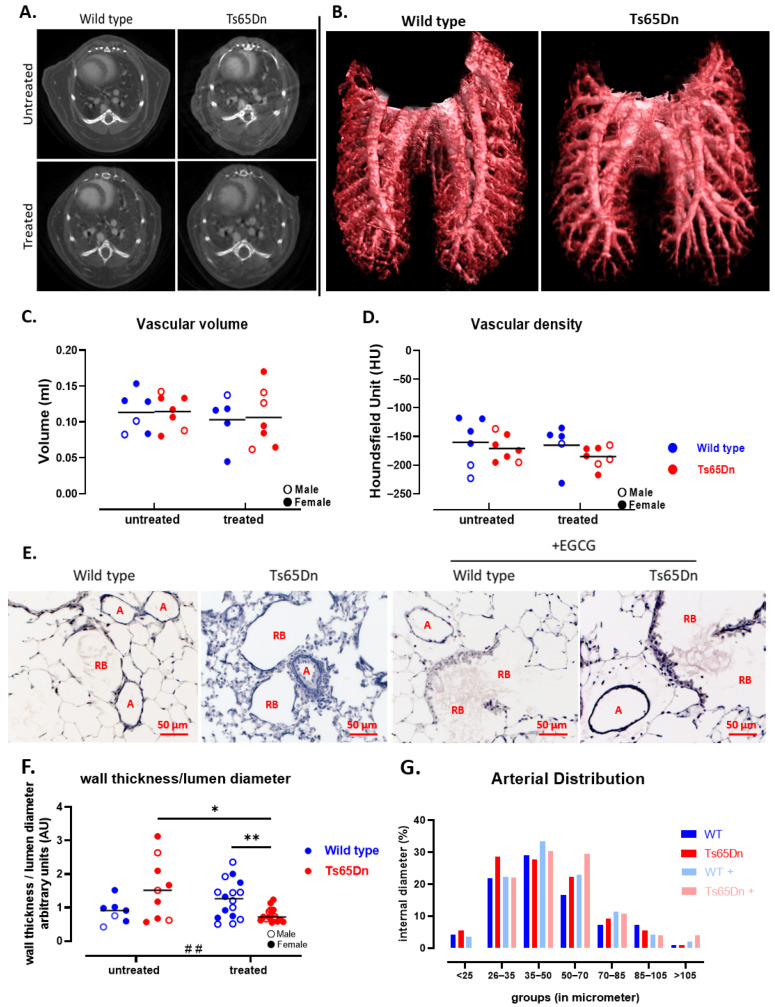
Visualization and quantification of the pulmonary vasculature in WT and Ts65Dn mice with and without GTE-EGCG administration. (**A**) Representative CE-µCT transversal images at treatment endpoint (PD180). (**B**) 3D reconstructions of macrovascular structures in lung ROI of WT and Ts65Dn mice. (**C**) Quantification of vascular volumes and (**D**) µCT-derived vascular density. Sample size; WT untreated n = 6 (2 males), Ts65Dn untreated n = 7 (2 males), WT treated n = 5 (1 males) and Ts65Dn treated n = 7 (3 males). (**E**) Histological representations of small pulmonary arteries using an Elastica van Gieson stain; A = arteries, RB = respiratory bronchiole, scale = 50 µm. (**F**) Arterial wall thickness measured on 12 cross-sectional arteries using histological sections. Sample size: WT untreated n = 7 (2 males), Ts65Dn untreated n = 9 (3 males), WT treated n = 16 (9 males), and Ts65Dn treated n = 16 (7 males). No outliers were identified. (**G**) Arterial internal diameter grouped according to size and expressed as percentage of total count (n = 12). Data are presented as individual values along with group means and distributions were analyzed by pairwise comparisons using unpaired *t*-tests (**C**,**D**) and Welch’s *t*-test (**F**) (* *p* < 0.05, ** *p* < 0.01). Interactions between genotype and treatment were analyzed using a two-way ANOVA (## *p* < 0.01) and males (open dot) and females (full dot) were presented accordingly.

To assess in vivo macrovascular changes in WT and trisomic mice, a CE-µCT scan was included at PD180. Qualitatively, the CE-µCT data did not reveal any overt genotype-induced structural vascular alterations (Figure 5A,B). Quantitative analysis of the CE-µCT-derived biomarkers demonstrated a similar vascular volume and vascular density between WT and Ts65Dn mice (Figure 5C,D), indicating no macrovascular alterations in the context of trisomy.

Motivated by the increased risk for developing PH in the DS population [4,49], we specifically examined microvascular changes and vascular wall morphology, with histological analysis (Figure 5E). There was no significant difference in arterial wall thickness or arterial distribution between WT and Ts65Dn mice (Figure 5F,G). Although Ts65Dn mice exhibited higher variability and a slight increase in wall thickness in the distal arteries, it did not reach significance when compared to WT mice (Figure 5F).

#### 3.3.2. GTE-EGCG Administration Decreases Arterial Wall Thickness in Ts65Dn Mice

We examined the impact of GTE-EGCG administration on the vascular component with CE-µCT. Macrovascular analysis revealed normal structure in both GTE-EGCG-treated and control groups. The CE-µCT-derived vascular parameters showed no variations in vascular volume or density among the four groups (Figure 5C,D). Microscopic examination of the microvasculature through histology of lung parenchyma demonstrated that GTE-EGCG administration normalized arterial wall thickness and vessel hypertrophy in Ts65Dn mice to levels comparable to WT mice, indicating genotype-specific changes (Figure 5F).

#### 3.3.3. Microstructural Analysis Reveals RV Hypertrophy in Ts65Dn Mice

**Figure 6:**
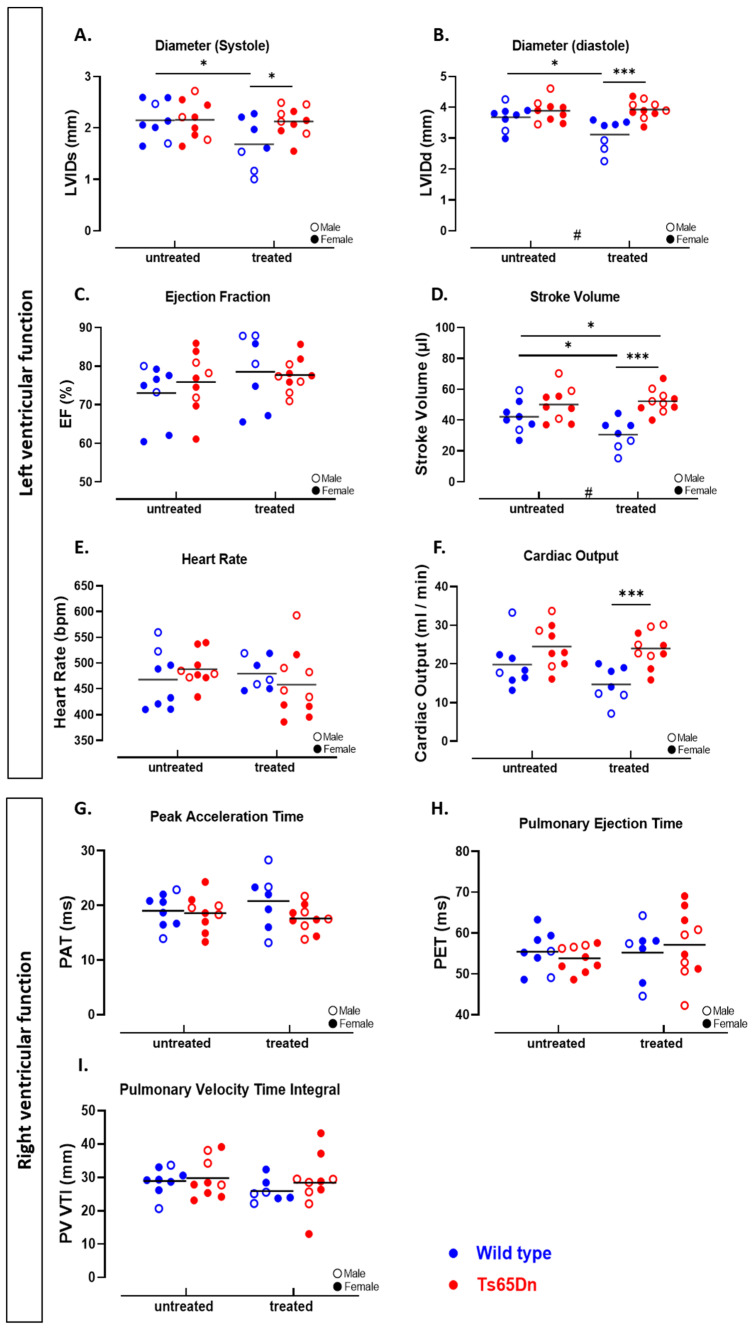
Functional analysis of cardiac parameters in WT and Ts65Dn mice with and without GTE-EGCG modulation. (**A**–**F**) Echocardiographic analysis of LV resulted in LV inner diameter at end-systole and end-diastole, allowing calculated ejection fraction and stroke volume. With corresponding heart rate, cardiac output was measured. (**G**–**I**) RV function was assessed by comparing pulmonary acceleration time (PAT), pulmonary ejection time (PET), and pulmonary valve velocity time integral (PV VTI) for WT and Ts65Dn mice with and without treatment. Data are presented as individual values along with group means and distributions compared by pairwise comparison including unpaired *t*-test (**A**,**B**,**D**–**F**,**I**), Welch’s *t*-test (**G**,**H**), or Kolmogorov–Smirnov test (**C**) (* *p* < 0.05, *** *p* < 0.001). Interactions between genotype and treatment were analyzed using a two-way ANOVA (# *p* < 0.05) and males (open dot) and females (full dot) were presented accordingly. The number of mice used were WT untreated n = 8 (2 males), Ts65Dn untreated n = 9 (3 males), WT treated n = 7 (3 males), and Ts65Dn treated n = 10 (5 males). No outliers were identified.

**Figure 7:**
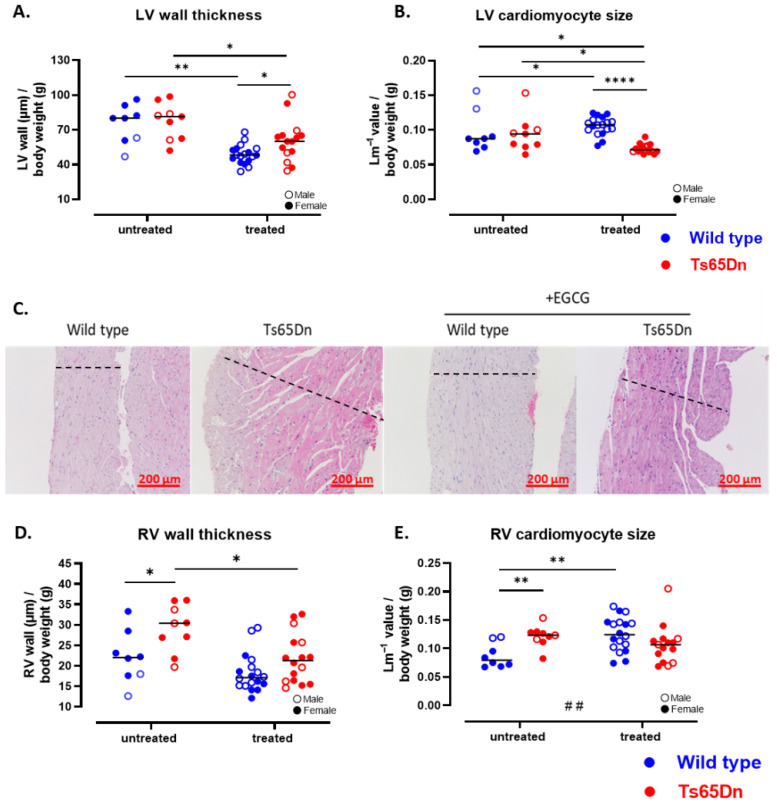
Structural analysis of LV and RV cardiac parameters in WT and Ts65Dn mice with and without GTE-EGCG modulation. (**A**) LV wall thickness, corrected for total body weight. (**B**) LV cardiomyocyte size, measured counting the number of transections within a reference line (Lm). (**C**) Representative histological images of RV wall thickness, dotted line indicating RV cross-section (scale bar = 200 µm). (**D**) RV wall thickness, corrected for total body weight. (**E**) RV cardiomyocyte size, calculated by Lm value. Data are presented as individual values along with group means and distributions compared by pairwise comparisons including Welch’s *t*-test (**A**), Mann–Whitney test (**D**,**E**), or Kolmogorov–Smirnov test (**B**) (* *p* < 0.05, ** *p* < 0.01, **** *p* < 0.0001). Interactions between genotype and treatment were analyzed using a two-way ANOVA (## *p* < 0.01) and males (open dot) and females (full dot) were presented accordingly. The number of mice used were WT untreated n = 8 (2 males), Ts65Dn untreated n = 9 (3 males), WT treated n = 18 (10 males), and Ts65Dn treated n = 16 (7 males). No outliers were identified.

To assess cardiac changes in the Ts65Dn mice, we performed echocardiography at treatment endpoint. LV function, including stroke volume, ejection fraction, and cardiac output, was similar for WT and Ts65Dn mice (Figure 6A–F). Both genotypes exhibited comparable left ventricular filling, mitral valve, and aortic valve function. We examined the microscopic structure of the heart and found comparable LV wall thickness in Ts65Dn and WT mice (Figure 7A). Analysis of individual LV cardiomyocyte size revealed no significant differences between the Ts65Dn and WT groups (Figure 7B).

Focusing on the RV, relevant to PH, echocardiographic analysis showed similar functional parameters, including pulmonary acceleration time, pulmonary ejection time, pulmonary vascular resistance, and pulmonary valve velocity time integral (Figure 6G–I). At the microstructural level, ex vivo histological analysis revealed a thicker RV wall in Ts65Dn mice compared to WT mice (Figure 7C,D). In addition, the size of the cardiomyocytes was larger in the Ts65Dn mice compared to the WT mice (Figure 7E) which may indicate RV hypertrophy.

#### 3.3.4. GTE-EGCG Administration Affects Both LV and RV Cardiac Function and Structure

We evaluated the impact of GTE-EGCG administration on cardiac function using echocardiography at treatment endpoint. LV analysis revealed significant changes in systolic and diastolic volumes in the WT mice treated with GTE-EGCG (Figure 6A,B). Although the ejection fraction was similar across all groups following GTE-EGCG administration (Figure 6C), stroke volume was decreased in the WT treated group compared to the WT untreated mice (Figure 6D). In contrast, GTE-EGCG had no effect on LV cardiac function in the trisomic mice (Figure 6A–F).

The interaction effect suggests a genotype-dependent response to GTE-EGCG administration on LV cardiac function (Figure 6D).

Similar to the LV measures on SV, microstructural analysis using histopathological measures showed a decrease in LV wall thickness in both WT and Ts65Dn treated groups (Figure 7A). However, the effect of GTE-EGCG on LV wall thickness was lower in the Ts65Dn compared to the WT treated mice. The WT treated mice also presented LV hypertrophy, as shown by an increase in cardiomyocyte size (Figure 7B). The Ts65Dn mice presented an opposite result (Figure 7B), thereby demonstrating a strong interaction between genotype and treatment effect.

Echocardiographic analysis of RV function showed no differences in functional measures as PAT, PET, and PV VTI was similar between the four groups (Figure 6G–I). Histological analysis revealed that GTE-EGCG administration decreases the RV wall thickness in Ts65Dn mice (Figure 7C,D). However, there was no difference in cardiomyocyte size between the trisomic untreated and trisomic treated groups (Figure 7E). Administration of GTE-EGCG to WT mice showed a similar wall thickness (Figure 7C,D) but a significant increase in cardiomyocyte size, indicating a drop in the number of cardiomyocytes in the RV wall (Figure 7E).

In summary, we characterized the cardiac and pulmonary vascular phenotype of Ts65Dn mice, revealing no macrostructural changes in pulmonary vasculature. However, microvascular analysis showed arterial hypertrophy in Ts65Dn mice, associated with signs of RV hypertrophy. GTE-EGCG administration normalized arterial thickening and reduced RV hypertrophy initiation in trisomic mice but not in WT mice which presented overall negative cardiac effects after GTE-EGCG treatment.

### 3.4. The Systemic and Pulmonary Immunological Status of Ts65Dn Mice and Effect of GTE-EGCG

#### 3.4.1. Ts65Dn Mice Have Less B-Cells

**Figure 8:**
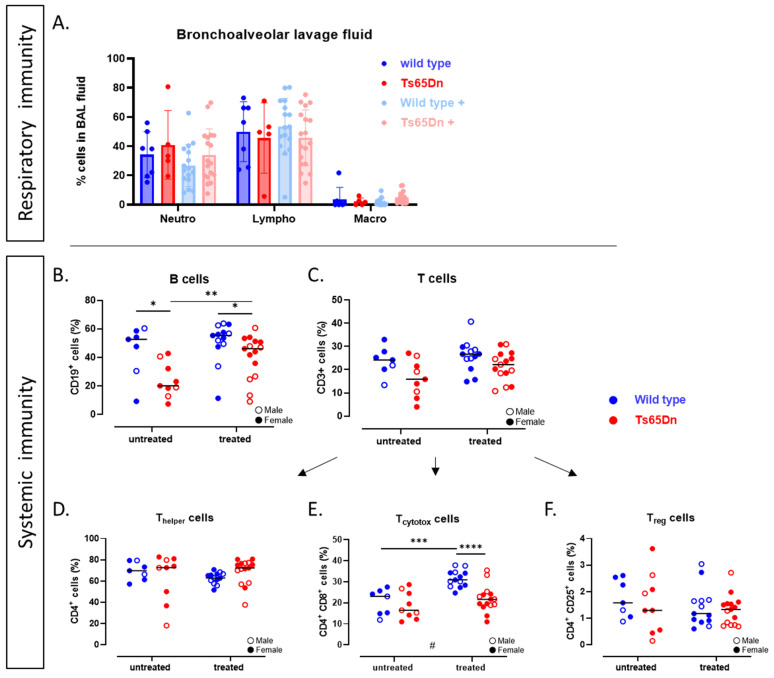
Systemic and pulmonary immunological analysis. (**A**) Relative proportions of neutrophils (Neutro), lymphocytes (Lympho), and macrophages (Macro) in bronchoalveolar lavage fluid. (**B**–**F**) Flow cytometric analysis of splenic lymphocytes: (**B**) CD19^+^ B lymphocytes (% of total cells), (**C**) CD3^+^ T lymphocytes (% of total cells), (**D**) CD3^+^CD4^+^ T helper lymphocytes (% of total T lymphocytes), (**E**) CD3^+^CD8^+^ cytotoxic T lymphocytes (% of total T lymphocytes), and (**F**) CD3^+^CD4^+^CD25^+^ regulatory T cells (% of total T lymphocytes). Bars show mean ± SD. All parameters are shown for each individual value, along with group means. Distributions were compared by pairwise comparisons including unpaired *t*-test (**C**,**E**), Welch’s *t*-test (**F**), Mann–Whitney test (**B**), or Kolmogorov–Smirnov test (**D**) (* *p* < 0.05, ** *p* < 0.01, *** *p* < 0.001, **** *p* < 0.0001); interactions between genotype and treatment were analyzed using a two-way ANOVA (# *p* < 0.05) and males (open dot) and females (full dot) were presented accordingly. Sample size: WT untreated n = 7 (2 males), Ts65Dn untreated n = 9 (3 males), WT treated n = 13 (6 males), and Ts65Dn treated n = 16 (7 males). No outliers were identified.

We assessed the immune status of Ts65Dn and WT mice to envisage potential immune differences and effect on the lung phenotype. Pulmonary immune analysis revealed similar cell counts of monocytes, lymphocytes, neutrophils, and eosinophils in BAL fluid (Figure 8A). Systemic immune analysis of the spleen showed a lower percentage of B-lymphocytes in Ts65Dn mice compared to WT (Figure 8B). T-lymphocyte numbers and differentiation into regulatory, cytotoxic, and helper T cells were similar between Ts65Dn and WT mice (Figure 8C–F).

#### 3.4.2. GTE-EGCG Administration Alleviates B-Cell Numbers in Ts65Dn Mice

To assess the persistent effects of GTE-EGCG administration on the immunological phenotype, we analyzed BAL fluid cell count. Results showed no significant changes in neutrophils, lymphocytes, and macrophages between WT and Ts65Dn mice (Figure 8A). GTE-EGCG administration did not affect total B-lymphocyte number in WT mice, but in Ts65Dn mice, the levels of B-cells were increased compared to the untreated trisomic group (Figure 8B) in the range of untreated WT levels, indicating a normalization of the number of B-cells. GTE-EGCG treatment resulted in a persistent increase in cytotoxic T-lymphocytes in WT mice, while no such effect was observed in Ts65Dn mice (Figure 8E).

In summary, our findings revealed a genotype effect, with significantly lower B-cell numbers and slightly lower T-cell numbers in Ts65Dn mice. These differences in B-cells were normalized by GTE-EGCG administration. Administration of GTE-EGCG showed drastic increase in levels of cytotoxic T-lymphocytes in the WT mice.

## 4. Discussion

Despite the cardiovascular and pulmonary abnormalities in individuals with DS, preclinical studies addressing these alterations remain scarce due to the lack of a well-characterized and consistent animal model. Our study aimed to fill this gap by characterizing cardiopulmonary development and immune phenotypes in Ts65Dn mice, and by evaluating how GTE-EGCG supplementation modulates these systems, including whether its effects persist after treatment discontinuation.

### 4.1. Genotype, but No Treatment Effects in Body Mass Development

When monitoring body weight as a proxy for body size, we observed that GTE-EGCG administration did not affect overall mass development. Trisomic mice in the treated groups weighed less than their euploid littermates, although this difference did not reach statistical significance in the untreated groups. This deviates from prior studies, reporting lower body weight in Ts65Dn mice [35,61,62], possibly due to genotype-independent, sex-specific differences [63], as most previous studies focused primarily on male mice [34,35,36,47,61]. While our study was not powered for a detailed analysis of sex-related differences, adult euploid males in the untreated mixed-sex cohort were generally heavier than their female trisomic littermates, aligning with previous reports. The limited number of male euploid mice in our study may explain the lack of statistical significance in weight differences in the untreated group, underscoring the need for future studies to include both sexes to better represent the heterogeneous nature of DS.

Besides body weight, body length is also relevant when considering body size. Our trisomic mice had shorter tibiae than their WT littermates [61], consistent with previous studies [64,65,66,67] and the shorter stature observed in humans with DS [68,69,70].

### 4.2. Insight into the Cardiopulmonary and Immunological Phenotype of Ts65Dn Mice

Our µCT analysis indicated no significant macrostructural alterations in Ts65Dn mice, indicating normal lung growth and structure. The semi-sterile housing conditions likely minimize exposure to infectious agents, reducing potential secondary effects of respiratory infections on lung development [3,71]. Consistent with previous reports [72], Ts65Dn mice exhibited an immature immune system, with significantly reduced B-cell counts and a trend toward lower T-cell counts. This aligns with the well-documented immunodeficiency in individuals with DS, who often present with reduced numbers of circulating B lymphocytes, diminished naïve T-cell pools, and impaired antibody responses [10,73,74,75]. In humans this immunological immaturity contributes to increased susceptibility to recurrent tract infections, higher hospitalization rates for pneumonia and bronchiolitis and reduced vaccine responsiveness. While we did not determine the direct impact of recurrent infections on lung development, the parallel reduction in B-cells in the Ts65Dn mice resembles that seen in individuals with DS and supports the model’s potential for preclinical studies of respiratory infections or post-vaccination responses in a trisomic context [76].

Signs of cardiopulmonary vulnerability were also observed [77,78]. Microvascular alterations, including increased vascular wall thickness and smaller lumen size, suggest a potential predisposition to PH, consistent with the notion that PH initially affects fragile (pre)capillaries and small arteries, progressively involving larger vessels in response to elevated pulmonary pressures [79]. While CE-µCT revealed no significant changes in large pulmonary arteries, vascular pruning or microvascular rarefaction cannot be excluded. Additionally, histopathological analysis revealed a significantly thicker RV wall in Ts65Dn mice, along with significantly larger cardiomyocytes indicative for RV hypertrophy, thereby, suggesting a compensatory response to potential pulmonary vascular remodeling. Together, these findings highlight the relevance of the Ts65DN model for studying early pathophysiological changes associated with PH and its use in developing preventive strategies.

Ts65Dn mice did not display severe CHD or other lethal congenital dysmorphologies, as indicated by the nearly equal distribution of genotypes after birth. Older studies have reported a proportional loss of trisomic offspring in late gestation, with a 20–40% transition of the marker chromosome due to fatal congenital defects such as CHD [20,80,81].

While Ts65Dn mice often show only mild or variable cardiac defects, Dp16 mice, another DS model, developed more consistent cardiopulmonary phenotypes with altered heart and lung biology [82], valuable for investigating CHD and related complications. While other models, such as the Ts1Cje and Tc1, exhibit more severe cardiac abnormalities including atrioventricular septal defects, these models also carry important limitations. In particular, the Tc1 model is mosaic and harbors multiple chromosomal rearrangements, duplication, and deletions which limit its generalizability to the human condition. Despite the clinical impact of CHD in DS, the underlying mechanisms of CHD remain incompletely understood. Progress has been made through the identification of specific dosage-sensitive genes such as DYRK1A, which have significantly advanced the genetic dissection of cardiac anomalies in DS [24,83]. Future studies may therefore benefit from integrating multiple complementary DS models to capture spectrum and molecular mechanisms of CHD in DS [84,85].

### 4.3. Is There an Effect of Prenatal GTE-EGCG Administration on Airway Development and Cardiac Function?

The use of GTE-EGCG in individuals with DS has been extensively studied for its potential cognitive and craniofacial effects [29,31,86,87]. However, its impact on the cardiopulmonary system remains unexplored, despite its involvement in antiangiogenic and antioxidative pathways [88,89,90]. In this study, we are the first to demonstrate that prenatal administration of GTE-EGCG affects airway development and cardiac function. GTE-EGCG administration leads to lung immaturity, evidenced by smaller overall lung size and decreased airway development as indicated by µCT biomarkers. Discontinuation of GTE-EGCG restores lung volume but not lung function, which remains reduced in both genotypes. Furthermore, GTE-EGCG treatment induces airway hyperreactivity in both WT and trisomic mice.

Remarkably, prenatal chronic GTE-EGCG administration decreased arterial wall thickness and RV wall thickness in trisomic mice, while no differences in RV wall thickness were observed in WT mice. However, WT mice showed an increase in RV cardiomyocyte size upon GTE-EGCG administration, indicating a hypertrophic cellular response despite stable wall thickness. In the LV, GTE-EGCG administration led to an increase in wall thickness and cardiomyocyte size in WT mice, consistent with a hypertrophic remodeling response. In contrast, Ts65Dn mice exhibited a decrease in LV wall thickness and cardiomyocyte size, demonstrating a strong interaction between genotype and treatment effect on cardiac microstructure.

This differential response may be due to the distinct gene expression profiles between WT and trisomic mice. In trisomic animals, GTE-EGCG may counteract gene overexpression linked to the extra chromosome, thereby exerting a corrective effect. In contrast, in WT mice with balanced gene expression, the same treatment may disrupt normal developmental pathways. The positive effect of GTE-EGCG on the RV could be a cause or consequence of its impact on the vasculature, requiring longitudinal structural analysis of the microvascular bed.

Our results suggest that GTE-EGCG administration positively affects the dysregulated pulmonary vascular system in Ts65Dn mice, potentially through its angiogenic properties [91,92]. However, variations in GTE-EGCG concentration or administration timing may activate different biomolecular pathways. Although EGCG is the most studied catechin and most abundant polyphenol in this GTE, the observed effects cannot be solely attributed to EGCG. Given that green tea extract is a complex mixture of polyphenols and also its metabolites, including EGC, EC, and ECG, are known to exert biological activity at low doses and which may contribute to the overall response. The diverse range of biological targets influenced by different catechins complicates mechanistic interpretation, warranting further research with individual pure substances to dissect the individual and combined contributions of these catechins and their downstream signaling effects, particularly in the context of angiogenesis and pulmonary development.

In the general population, recurrent lung infections have been associated with impaired pulmonary vascular maturation [93]. Thus, the effect of GTE-EGCG on the vasculature may directly or indirectly involve rescuing immune cell maturation. Our findings indicate that GTE-EGCG administration restored B lymphocyte populations in Ts65Dn mice, suggesting a modulatory effect on immune cells. Additionally, we observed an increase in cytotoxic T lymphocytes (CD8+ T cells), particularly in WT mice, indicating that GTE-EGCG influences both adaptive immune cell populations and potentially inflammatory tone. These results align with previous studies demonstrating the immunomodulatory potential of different concentrations of EGCG, promoting B-cell and T-cell proliferation in a murine leukemia model [94]. These findings underscore GTE-EGCG’s capacity to modulate immune responses, where these may be interpreted as potentially unwanted immune activation in WT mice.

The variations in GTE-EGCG doses and administration routes used across studies, as well as the unknowns regarding the effects of the various polyphenol metabolites, different administration routes or preclinical model make it very challenging to compare outcomes across studies [33,35]. Also, within a single study, administering GTE-EGCG over a prolonged period spanning embryonic to adult development such as ours, bioavailability of GTE-EGCG to different organs is more than likely to vary over time and probably even between genotypes when considering potential developmental differences in, for example, the blood–brain barrier. This complexity makes it virtually impossible to establish a dose–response relationship for organ-system specific modulatory effects. Indeed, when providing EGCG to developing pups via the pregnant dams’ drinking water, EGCG crosses the placental barrier, at a concentration of catechins about 10 times lower compared to the maternal plasma concentration [95]. Low concentrations of EGCG are also present in the milk and plasma of pups from PD1 to PD7 [96]. After weaning, the mice were given drinking water with a same concentration of GTE-EGCG as their mothers received. Based on these data and measures of water intake, one can only estimate the dose of EGCG that was eventually delivered to the mice in each developmental stage. For post-weaning treatment, administration via oral gavage may be considered for being favorable in order to standardize the daily EGCG dose intake, but needs to be offset against the daily stress that affects experimental outcomes [34,35].

Importantly, the treatment window applied in this study (starting at E9 in mice) does not have a direct human equivalent and cannot be replicated in clinical practice. This timepoint was selected because it corresponds to the initial formation of the trachea and lung buds [97], and also marks the onset of *Dyrk1A* expression in the developing mouse embryo. Further investigation into the potential differential effects of treatments across organ systems and developmental could help establish concentration- and time-dependent guidelines for safe and effective administration.

### 4.4. Limitations

While this study provides important insights into the cardiopulmonary and immunological phenotype of the Ts65Dn mouse model, several limitations need to be considered. First, the Ts65Dn model represents a partial trisomy for approximately two-thirds of the genes orthologous to human chromosome 21 [15,16,17], and carries 46 non-Hsa21 genes, which can influence the phenotype and complicate the direct translation of results to human DS [21,22]. Phenotypic drift and strain variability have been documented among different Ts65Dn strains [47], and may contribute to variability between reports. Such variability underscores the importance of careful model selection and reporting. Additionally, more recent genetically developed models of DS, such as the Ts66Yah and TcMAC21, may provide additional insights into the condition [23,24,25,98]. The use of multiple models will be crucial in capturing the disorder’s full spectrum of effects across different organ systems and phenotypes [99].

Second, the relatively small sample size limits our ability to detect potential genotype-by-sex interactions in detail. Although we observed trends in sex-specific differences, the study was not powered for detailed sex-based analysis, which restricts the generalizability of the findings [34,36]. As the male and female mice differed particularly in body weight, we corrected for body weight where applicable to reduce the confounding impact of sex-based size differences on phenotypic outcomes. Although the role of sex in organ development and disease progression, particularly in Down syndrome, has been increasingly recognized in human studies [98], studies exploring sexual dimorphism in the Ts65Dn model remain scarce, and most prior work has focused on males only. Our aim was to provide a broad overview of the treatment effects and phenotypic features of Ts65Dn mice across systems, relevant for both sexes. Where we found evidence for sex differentiation in the phenotype, follow-up studies can be explicitly designed to investigate sexual dimorphism, using sex-balanced cohorts powered for such analyses.

While the effects of the treatment and intervention were analyzed across different developmental stages, the variability in bioavailability and lack of precise individual dosing due to ad libitum administration via drinking water complicate the establishment of a clear dose–response relationship at each developmental stage, during pregnancy and weaning stages. The choice to administer GTE-EGCG via drinking water was intentional, as it allowed for long-term, low-stress exposure, albeit at the cost of dosing precision. While EGCG is the most abundant catechin in green tea extract, other polyphenols and metabolites may have a contribution to the observed effects. Given the complex pharmacokinetics of GTE-EGCG [96], future studies should refine dosing strategies and perform a dose-comparison of EGCG using more controlled delivery methods to better assess its therapeutic potential. Nevertheless, the effect size of our study exceeds the potential variability thereby introduced, further underscoring robustness of our observations.

Finally, while we observed pronounced alterations in B- and T-cell populations, we did not perform functional assays (e.g., infection challenge or vaccination studies) to directly validate how these immune changes affect host defense.

Importantly, although GTE-EGCG is a freely available dietary supplement, our findings emphasize the need for caution when considering its use during early development. Our results highlight beneficial effect but also serious adverse effects certain organs systems. These findings should therefore be interpreted within the context of preclinical mechanistic research rather than as translatable treatment guidelines.

## 5. Conclusions

This study characterizes the cardiopulmonary and immunological alterations in Ts65Dn mice, demonstrating RV hypertrophy, narrowed blood vessels and reduced B-cell counts, supporting their use in preclinical immune respiratory research. Prenatal GTE-EGCG administration induces transient lung immaturity, reduced inspiratory capacity, and airway hyperreactivity, while also normalizing arterial and RV wall thickness and improved hematopoiesis with increased B-cell lymphocytes. These findings provide a holistic multiorgan framework to evaluate modulatory agents in DS models, while highlighting that GTE-EGCG exerts both beneficial and adverse effects that must be carefully weighed, especially given its unrestricted availability as a readily accessible dietary supplement.

## Data Availability

Data presented in this study is contained within the article and supplementary material. Further inquiries can be directed to the corresponding author.

## Supplementary Materials

The following supporting information can be downloaded at: https://www.mdpi.com/article/10.3390/pharmaceutics17111366/s1, Video S1: Lung parenchyma_wild type; Video S2: Lung parenchyma_wild type treated; Video S3: Lung parenchyma_Ts65Dn treated; Table S1: Overview on offspring of the different litters including mother identity, litter number, pup number, genotype, treatment regimen, sex and body weight at PD3; Table S2: Overview normality, homoscedasticity and statistical test performed for pairwise comparison per parameter If one of the four mice groups was not normally distributed or one pairwise comparison was not homoscedastic, the variable was considered as not normally distributed and/or not homoscedastic.
