# Supplementary material for: Cardiopulmonary and Immune Alterations in the Ts65Dn Mouse Model of Down Syndrome and Modulation by Epigallocatechin-3-Gallate-Enriched Green Tea Extract
Source: Pharmaceutics. 2025 Oct 22;17(11):1366. doi: 10.3390/pharmaceutics17111366 (PMC12655474; doi:10.3390/pharmaceutics17111366)
Supplement: Supplementary file 1 [file pharmaceutics-17-01366-s001.zip › Pharmaceutics_3541092_Supplemental Table S2.pdf]

Supplemental Table S2: overview normality, homoscedasticity and statistical test performed for pairwise comparison per parameter. If one of the four mice groups was not normally distributed or one pairwise comparison was not homoscedastic, the variable was considered as not normally distributed and/or not homoscedastic.

	Figure	Parameter	Passed normality test? (shapiro	Equal SD according to F-test?	Test performed
body growth	2A	PD3	No	Yes	Mann-Whitney
		PD180	No	No	Kolmogorov -Smirnov
		PD210	No	Yes	Mann-Whitney
micro-CT - TLV	2D	PD3	No	No	Kolmogorov-Smirnov
		PD29	Yes	No	Welch's t-test
		PD180	No	No	Kolmogorov-Smirnov
		PD210	Yes	Yes	t-test
micro-CT - TLD	2E	PD3	Yes	Yes	t-test
		PD29	No	No	Kolmogorov-Smirnov
		PD180	No	No	Kolmogorov-Smirnov
		PD210	Yes	Yes	t-test
micro-CT - ALV	2F	PD3	No	No	Kolmogorov-Smirnov
		PD29	No	No	Kolmogorov-Smirnov
		PD180	Yes	No	Welch's T-test
		PD210	Yes	Yes	t-test
lung airspace	3B	PD210	Yes	Yes	t-test
lung function					
IC	4A	PD210	No	No	Kolgomorov-Smirnov
Rn	4B	PD210	No	No	Kolgomorov-Smirnov
H	4C	PD210	Yes	No	Welch's t-test
FVC	4D	PD210	No	No	Kolgomorov-Smirnov
$FEV_{0,1}$ / FVC	4E	PD210	No	No	Kolgomorov-Smirnov
$FEV_{0,1}$	4F	PD210	No	No	Kolgomorov-Smirnov
airway reactivity - AUC	4H	PD210	Yes	Yes	t-test
Vascular volume	5C	PD180	Yes	Yes	t-test
Vascular density	5D	PD180	Yes	Yes	t-test
Arterial wall / lumen	5F	PD210	Yes	No	Welch's t-test
LV - systolic diameter	6A	PD180	Yes	Yes	t-test
LV - diastolic diameter	6B	PD180	Yes	Yes	t-test
LV- EF	6C	PD180	No	No	Kolgomorov-Smirnov
LV - SV	6D	PD180	Yes	Yes	t-test
LV- HR	6E	PD180	Yes	Yes	t-test
LV CO	6F	PD180	Yes	Yes	t-test
RV - PAT	6G	PD180	Yes	No	Welch's t-test
RV - PET	6H	PD180	Yes	No	Welch's t-test
RV - PVTI	6I	PD180	Yes	Yes	t-test
LV wall thickness	7B	PD210	Yes	No	Welch's t-test
LV cardiomyocyte size	7C	PD210	No	No	Kolgomorov-Smirnov
RV wall thickness	7D	PD210	No	Yes	Mann-Whitney
RV cardiomyocyte size	7E	PD210	No	Yes	Mann-Whitney
systemic - B-cells	8B	PD210	No	Yes	Mann-Whitney
systemic - T-cells	8C	PD210	Yes	Yes	t-test
systemic - T_{helper}-cells	8D	PD210	No	No	Kolgomorov-Smirnov
systemic - $T_{cytotox}$-cells	8E	PD210	Yes	Yes	t-test
systemic - T_{reg}-cells	8F	PD210	Yes	No	Welch's t-test
